# Integrating AI detection and language models for real-time pest management in Tomato cultivation

**DOI:** 10.3389/fpls.2024.1468676

**Published:** 2025-02-21

**Authors:** Yavuz Selim Şahin, Nimet Sema Gençer, Hasan Şahin

**Affiliations:** ^1^ Bursa Uludağ University, Faculty of Agriculture, Department of Plant Protection, Bursa, Türkiye; ^2^ Bursa Technical University, Faculty of Engineering and Natural Sciences, Department of Industrial Engineering, Bursa, Türkiye

**Keywords:** pest detection, precision agriculture, ChatGPT, YOLOv8, sustainable agriculture

## Abstract

Tomato (*Solanum lycopersicum* L.) cultivation is crucial globally due to its nutritional and economic value. However, the crop faces significant threats from various pests, including *Tuta absoluta*, *Helicoverpa armigera*, and *Leptinotarsa decemlineata*, among others. These pests not only reduce yield but also increase production costs due to the heavy reliance on pesticides. Traditional pest detection methods are labor-intensive and prone to errors, necessitating the exploration of advanced techniques. This study aims to enhance pest detection in tomato cultivation using AI-based detection and language models. Specifically, it integrates YOLOv8 for detection and segmentation tasks and ChatGPT-4 for generating detailed, actionable insights on the detected pests. YOLOv8 was chosen for its superior performance in agricultural pest detection, capable of processing large volumes of data in real-time with high accuracy. The methodology involved training the YOLOv8 model with images of various pests and plant damage. The model achieved a precision of 98.91%, recall of 98.98%, mAP50 of 98.75%, and mAP50-95 of 97.72% for detection tasks. For segmentation tasks, precision was 97.47%, recall 98.81%, mAP50 99.38%, and mAP50-95 95.99%. These metrics demonstrate significant improvements over traditional methods, indicating the model’s effectiveness. The integration of ChatGPT-4 further enhances the system by providing detailed explanations and recommendations based on detected pests. This approach facilitates real-time expert consultation, making pest management accessible to untrained producers, especially in remote areas. The study’s results underscore the potential of combining AI-based detection and language models to revolutionize agricultural practices. Future research should focus on training these models with domain-specific data to improve accuracy and reliability. Additionally, addressing the computational limitations of personal devices will be crucial for broader adoption. This integration promises to democratize information access, promoting a more resilient, informed, and environmentally conscious approach to farming.

## Introduction

Tomato (*Solanum lycopersicum* L.) is a globally significant vegetable crop, essential for both nutritional value and economic stability. However, tomato cultivation faces substantial threats from various pests. Key pests include *Tuta absoluta* (Lepidoptera: Gelechiidae), which has a significant socioeconomic impact in Eastern Africa due to its widespread distribution and increased costs and pesticide use among farmers (Pereyra and Sánchez, 2006; Shaltiel-Harpaz et al., 2015; Aigbedion-Atalor et al., 2019). *Helicoverpa armigera* (Lepidoptera: Noctuidae) is another critical pest, highlighting the low adoption of biological control measures and underscoring the need for improved farmer knowledge and extension programs (Balipoor and Ommani, 2014). *Leptinotarsa decemlineata* (Coleoptera: Chrysomelidae) and *Bemisia tabaci* (Hemiptera: Aleyrodidae) also pose substantial threats. *Myzus persicae* (Hemiptera: Aphididae), along with *Dolycoris baccarum* (Hemiptera: Pentatomidae), *Phyllotreta* spp. (Coleoptera: Chrysomelidae), and *Nezara viridula* (Hemiptera: Pentatomidae), further complicate tomato cultivation. Additionally, *Tetranychus urticae* (Trombidiformes: Tetranychidae) is a significant allergen, particularly among greenhouse workers and asthmatics living near orchards (Jee et al., 2000). *Frankliniella occidentalis* (Thysanoptera: Thripidae) is another pest impacting tomato crops. Insecticide use patterns among tomato farmers in Ghana reveal a mix of recommended and non-recommended, persistent insecticides, highlighting the need for better regulation and education (Danquah et al., 2010).

Efficient and timely identification of pests is essential for maintaining crop health and optimizing yield. Traditionally, this process has relied heavily on human observation, which is labor-intensive, time-consuming, and susceptible to errors (Danquah et al., 2010). Artificial Intelligence (AI) models, which use algorithms and computational power to simulate human intelligence, offer a promising alternative. There are various types of AI models for data processing: some models process images by converting them into matrices (detection models), while others process text by converting characters or tokens into vectors (language models) (Vaswani et al., 2017). Detection models, such as Mask R-CNN, Faster R-CNN, SSD, and YOLO (You Only Look Once), provide rapid and accurate pest detection, significantly reducing the need for manual labor and enhancing precision. They are capable of processing large volumes of data in real-time, thereby greatly improving agricultural efficiency and sustainability (Liu and Wang, 2020; Swinburne et al., 2022; Jin et al., 2022; Wen et al., 2022; Rajamohanan and Latha, 2023).

YOLO excels due to its real-time processing, high detection accuracy, and versatility in both detection and segmentation tasks. Unlike traditional AI-based pest management systems, this study introduces a novel integration of real-time detection with YOLOv8 and language-based decision support via ChatGPT-4, offering both precision in pest detection and actionable, context-specific recommendations for farmers. This combination allows not only for accurate detection but also for informed decision-making, making the system accessible and practical for real-world agricultural applications. By reducing the reliance on manual expertise and providing timely insights, this system improves both the efficiency and sustainability of pest management practices. It is particularly effective for detecting small, densely packed objects like agricultural pests, making it ideal for real-time applications (Redmon et al., 2016). Its adaptability to various scales and high mean Average Precision (mAP) scores further justify its use in training and detecting agricultural pests, effectively managing multiple pest species with diverse morphologies (Yang et al., 2020; Hashimoto et al., 2020). These features make YOLO an excellent choice for pest detection and segmentation in this study.

However, while detection models like YOLO have the potential to analyze pests more accurately and quickly than humans, they lack the capability to interpret the findings and provide actionable recommendations to farmers (Swinburne et al., 2022; Jin et al., 2022). This gap, which requires knowledge and experience, can be filled by language models. Language models, like ChatGPT, are a type of AI designed to understand and generate human language. They process data by converting characters into vectors, which allows the model to recognize and predict patterns in text (Vaswani et al., 2017). ChatGPT-4, developed by OpenAI, was trained on approximately 1.3 trillion tokens, providing it with a vast knowledge base (Rao et al., 2023). Therefore, while YOLO is used for accurate and real-time pest detection, ChatGPT was chosen as the language model for this study due to its extensive training and ability to generate relevant, insightful responses to interpret the detected tomato pests.

Accurate and real-time identification of agricultural pests necessitates education, knowledge, and experience (Swinburne et al., 2022; Danquah et al., 2010). Once pests are detected, it is essential to have detailed information about them to devise effective management strategies (Balipoor and Ommani, 2014; Aigbedion-Atalor et al., 2019). Accessing this information can be time-consuming and costly. However, language models can provide detailed commentary on detected pests in agricultural applications, thus informing farmers who may lack expertise. By facilitating access to accurate information and analyzing large datasets more quickly than humans, these models can save time and costs while enhancing the quality of education. In this study, detection models and language models are integrated through an API (Application Programming Interface, a set of rules and protocols for building and interacting with software applications, allowing different systems to communicate and share data) to analyze and interpret pest data, providing a valuable guide for future similar research endeavors.

## Materials and methods

### Definition of the research area and dataset

Turkey is one of the top five tomato-producing countries in the world. About 10% of Turkey’s tomato production occurs in Bursa, where tomatoes were the most produced vegetable in 2020, with 13.2 million tons (Kumbasaroğlu et al., 2021). This study was conducted from March 2022 to September 2023 in Bakirköy village, located in the Karacabey district of Bursa province in the northwest of Turkey, lying between latitudes 40°7’17.53”N and 40°10’40.36”N and longitudes 28°21’14.12”E and 28°26’2.37”E. Field campaigns were conducted from June to July 2023. The site covers an area of 47.16 km², and a total of 96 tomato fields were investigated. The identification of pests observed in the field photographs was carried out according to the morphological diagnostic keys available in the literature (Blackman and Eastop, 2000; Hoebeke and Carter, 2003; Desneux et al., 2010; Ashbrook et al., 2022; Li et al., 2023).

Detection models excel in identifying the presence and location of pests quickly and efficiently (Barbedo, 2016). However, segmentation models are more suitable when detailed morphological features or comprehensive damage maps are necessary (Arockia et al., 2023). The YOLOv8 model integrates both detection and segmentation capabilities. In this study, the yolov8s.pt model was employed for detection tasks, while the yolov8n-seg.pt model was utilized for segmentation tasks. The images used for detection and segmentation in this study encompass various pests and damage types affecting tomato crops. These include *Dolycoris baccarum* (*Hemiptera: Pentatomidae*), *Phyllotreta* spp. (*Coleoptera: Chrysomelidae*), *Nezara viridula (Hemiptera: Pentatomidae), Myzus persicae (Hemiptera: Aphididae), Bemisia tabaci (Hemiptera: Aleyrodidae), Leptinotarsa decemlineata (Coleoptera: Chrysomelidae), Tuta absoluta (Lepidoptera: Gelechiidae), Helicoverpa armigera (Lepidoptera: Noctuidae), Liriomyza bryoniae (Diptera: Agromyzidae) damage, Frankliniella occidentalis (Thysanoptera: Thripidae)* damage, and *Tetranychus urticae (Trombidiformes: Tetranychidae)* damage. These pests and damage types were systematically photographed and used to train the YOLOv8 model for accurate detection and segmentation tasks, aiming to enhance the model’s ability to identify and manage multiple pest species effectively.

From March 2023 to September 2024, high-resolution images of tomato plant diseases and pests were captured using a Canon EOS 700D camera with a resolution of 768 × 1024 pixels. To ensure consistency in image quality, all photographs were taken using cameras set to identical resolution settings. Images were taken at distances of 1 meter and 0.2 meters from the leaves, from various angles (Yong et al., 2020). A comprehensive dataset of over 1,000 images was compiled for each pest, documenting different angles and features. These images were then divided into three subsets: 80% for training, 18% for validation, and 2% for testing, as outlined in **Table 1**.

**Table 1:** Distribution of the image dataset for model training.

Method	Image Type	Total Images	Training %80	Validation %18	Test %2
**Detection**	Pest adult images	7000	5600	1260	140
	Pest nymph images	2000	1600	360	40
	Pest larva images	2000	1600	360	40
**Total Images**		11000	8800	1980	220
**Segmentation**	Tomato leaf images	5000	4000	900	100
	Tomato fruit images	4000	3200	720	80
**Total Images**		9000	7200	1620	180

### Data preprocessing techniques and applications

Data preprocessing refers to a series of steps undertaken to prepare raw data for analysis or modeling. It is commonly used in data mining, machine learning, statistics, and data analysis to address data deficiencies, noise, and inconsistencies, thereby enabling more effective analysis (Şahin and Topal, 2016; Atalan et al., 2022; Baştürk and Şahin, 2022). For image processing models, preprocessing the dataset involves three steps: image labeling, resizing, and augmentation. Augmentation provides a large amount of training data to learn features and achieve accurate classification on unseen data, preventing issues like overfitting and poor generalization (Redmon and Farhadi, 2018; Rubanga et al., 2020; Güven and Şahin, 2022).

In this study, Python (version 3.11.8) was used for data preprocessing due to its extensive library ecosystem. To enhance the quality of model training, the original images taken under field conditions were augmented using the OpenCV library. During augmentation, transformations were applied to each image, including rotation, cropping, flipping, adding noise, adjusting lighting, and zooming out (Shorten and Khoshgoftaar, 2019). All images were resized to 600 × 600 pixels as required for model training (He et al., 2017). The analyses were conducted in the Spyder IDE, part of the Anaconda distribution, which offers various libraries for scientific computing and data science (Şahin et al., 2020; Yılmaz et al., 2021). Prior to any augmentation, the dataset was divided into training, validation, and test sets (80%, 18%, and 2%, respectively) to ensure that no data leakage occurred during the augmentation process. Augmentation was only applied to the training set to avoid introducing artificial examples into the validation and test sets, which could result in overly optimistic performance estimates (Shorten & Khoshgoftaar, 2019). Specifically, transformations such as rotation, cropping, flipping, adding noise, adjusting lighting, and zooming out were applied only to the training data after the initial dataset split. Labeling was performed using the LabelMe tool (https://github.com/wkentaro/labelme), with two main approaches: pixel-based segmentation for precise boundary definitions and rectangular bounding for approximate location and size.

### Model setup and training

**Figure 1:**
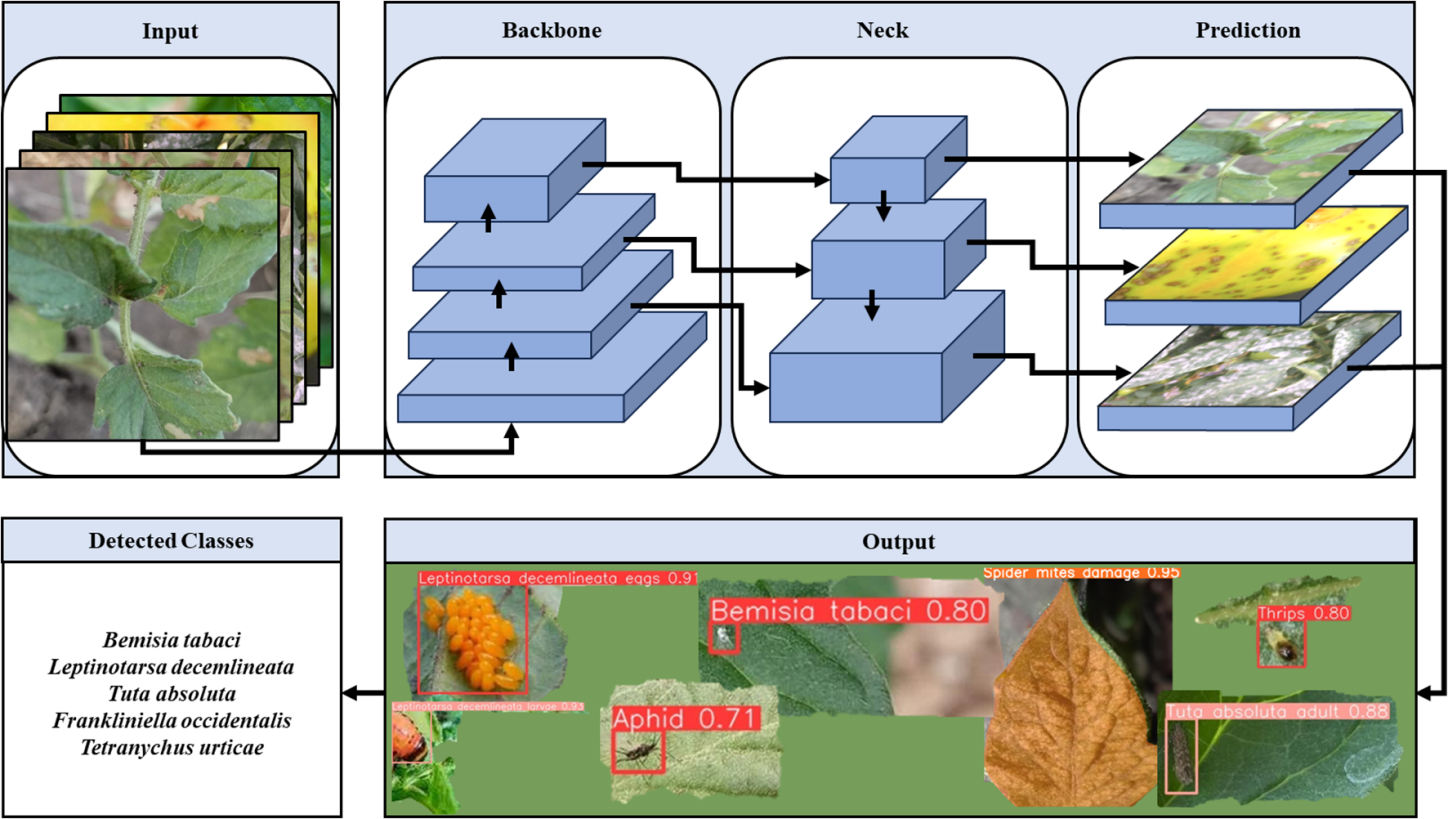
YOLO Framework used in this study. Input: Raw images fed into the model. Backbone: Extracts features from images. Neck: Combines and enhances features. Prediction: Predicts pests’ presence and location. Output: Provides detection and segmentation results.

YOLOv8 was selected for this study due to its superior speed and efficiency compared to slower yet more accurate models like Faster R-CNN, making it particularly well-suited for real-time agricultural pest detection, where timely decisions are essential for effective pest management (Tang et al., 2021). The YOLOv8 models were trained using the ultralytics library for model loading and training, and the google.colab library for accessing the dataset via Google Drive. Training parameters included over 100,000 epochs (with patience set to 50 to prevent overfitting), a batch size of 16, and an image size of 640 (**Table 2**). The model’s hyperparameters, including the number of epochs, batch size, and learning rate, were optimized through an iterative process. Early stopping (patience) was employed to prevent overfitting, while cross-validation was used to fine-tune the learning rate and batch size. The optimal values for these parameters were selected based on the model’s performance on the validation set, ensuring robustness and preventing overfitting. Training was conducted on Google Colaboratory, utilizing an Intel Xeon CPU, 12.68 GB RAM, and a Tesla K80 GPU. Both detection and segmentation models were trained in Python on a custom dataset. Instance segmentation models were chosen to precisely identify damage caused by multiple pest species on tomato plants, which is crucial for accurately identifying specific damages on leaves and fruits (Mirhaji et al., 2021; Zhang et al., 2023a, 2023). The YOLO framework used in this study is illustrated in **Figure 1**.

**Table 2:** Key parameters were set in Google Colab for the training of the Ultralytics YOLOv8.

Task	Mode	model	epochs	batch	imgsz	patience
segment	train	yolov8s.pt	10000	16	640	50
detect	train	yolov8n-seg.pt	10000	16	640	50

### Model evaluation methodology and testing process

The model’s generalization capability was assessed using a pre-allocated dataset: 80% for training, 18% for validation, and 2% for testing. Key performance metrics, including precision, recall, mAP50, and mAP50-95, were calculated during training on both training and validation datasets. Detection and segmentation performances were evaluated at various IOU thresholds using precision (P), recall (R), and mean average precision (mAP). The mAP50 metric refers to the mean average precision at a 50% IOU threshold, indicating the accuracy of the model in identifying objects with at least 50% overlap with ground truth labels. On the other hand, mAP50-95 averages precision over IOU thresholds from 50% to 95%, offering a more comprehensive view of the model’s accuracy across different overlap scenarios, which is particularly important in agricultural contexts where pests may be occluded or vary in size (Li et al., 2023). These metrics provide insights into the model’s ability to handle various sizes and overlaps in real-world agricultural environments. Loss metrics—box_loss, cls_loss, and dfl_loss—were analyzed to identify areas for improvement. The confusion matrix summarized predictions across classes, highlighting correct and incorrect classifications. This comprehensive analysis provided a clear understanding of the model’s strengths and weaknesses. Metrics P, R, mAP50, and mAP50-95 are defined by **Table 3**.

**Table 3:** Formulas of key performance metrics for evaluating YOLO models in object detection.

Performance metrics	Formula
Precision (P)	$\frac{TP}{\left(TP+FP\right)}$
Recall (R)	$\frac{TP}{\left(TP+FN\right)}$
mAP50	$\frac{1}{Q}\sum_{q-1}^{Q}P\left(\text{Rq}\right)$
mAP50-95	$\frac{1}{Q}\sum_{i-5}^{95}P\left(Rq\right)\frac{1}{Qi}\sum_{q-1}^{Qi}P\left(Rq,i\right)$

TP (True Positives): The count of correct positive predictions. FP (False Positives): The count of incorrect positive predictions (actual negatives predicted as positives). FN (False Negatives): The count of incorrect negative predictions (actual positives predicted as negatives). Q: Number of query points. P(Rq): Interpolated precision at recall level Rq.

### Prompt creation and OpenAI GPT-4 integration

**Figure 2:**
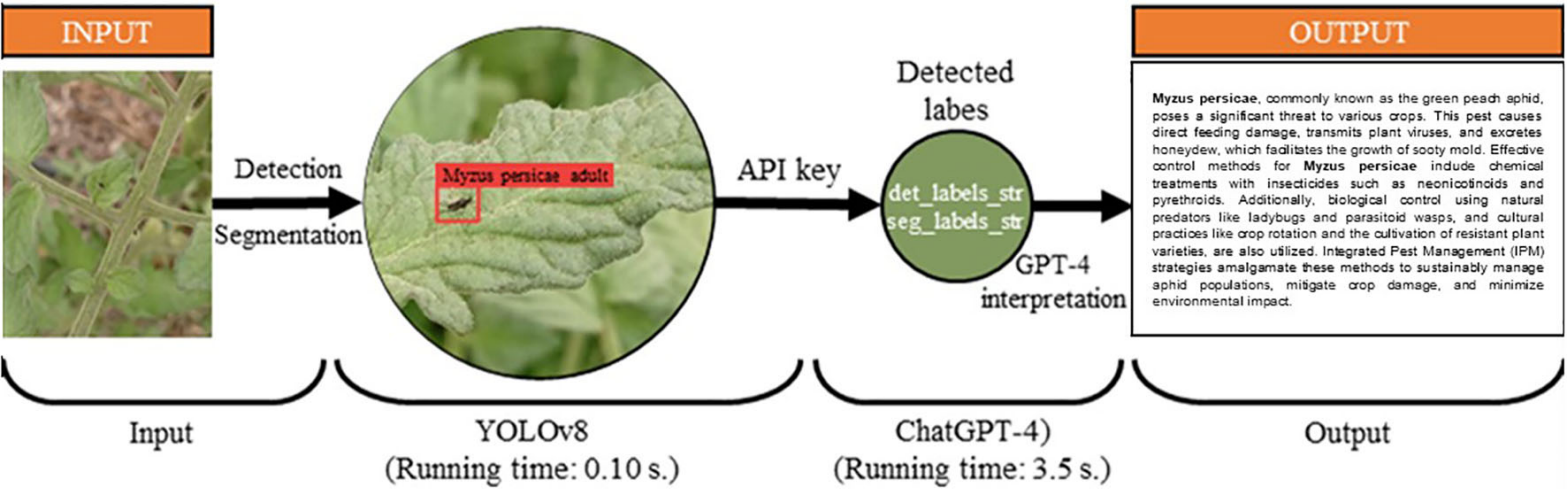
ChatGPT-4 integration process.

The study used Python and open-source libraries to integrate detection models with OpenAI’s GPT-4 via an API key. Initially, models identified trained objects, which were then linked to GPT-4. A good prompt should be clear, specific, and provide context to guide the AI’s response. Labels were defined as ‘det_labels_str’ and ‘seg_labels_str’. The prompt used in the study was: prompt_str = f”Could you provide a detailed explanation, in academic English, on the methods for controlling {det_labels_str} or {seg_labels_str} and the potential damage they can inflict on related plants, including preventative measures and integrated pest management strategies?”. Text outputs, limited to 250-400 tokens, were visualized with detection results. A ten-step coding sequence enabled the simultaneous operation of segmentation and detection models (**Table 4**). The workflow, from image capture to ChatGPT-4 output, is depicted in **Figure 2**.

**Table 4:** Integration of Ultralytics YOLO and OpenAI GPT-4 using API key.

Steps		Description
1	Model Loading	Pre-trained segmentation and detection models are loaded using the YOLO framework.
2	Image Loading	The image to be analysed has been uploaded.
3	Segmentation	The segmentation model is run on the image, and the predicted masks are obtained.
4	Application of Masks	The predicted masks are applied to the original image to identify specific areas.
5	Detection	The detection model is executed on the combined image, predicting bounding boxes and class names for objects.
6	Visualization of Results	Both segmentation and detection outcomes are visualized and saved for further analysis.
7	Labelling	The segmentation and detection results are converted into labels and formatted as strings.
8	Integration with Natural Language Processing	By connecting to OpenAI’s GPT-4 through its API, a question is formulated regarding the detected labels, and a response is subsequently obtained.
9	Prompt Creation for GPT-4	prompt_str = f”Could you provide a detailed explanation, in academic English, on the methods for controlling {det_labels_str} or {seg_labels_str} and the potential damage they can inflict on related plants, including preventative measures and integrated pest management strategies?”
10	Combination and Visualization of Results	The detection results and the text response from GPT-4 are visualized and saved.

## Results

### Training and validation loss graphs

**Figure 3:**
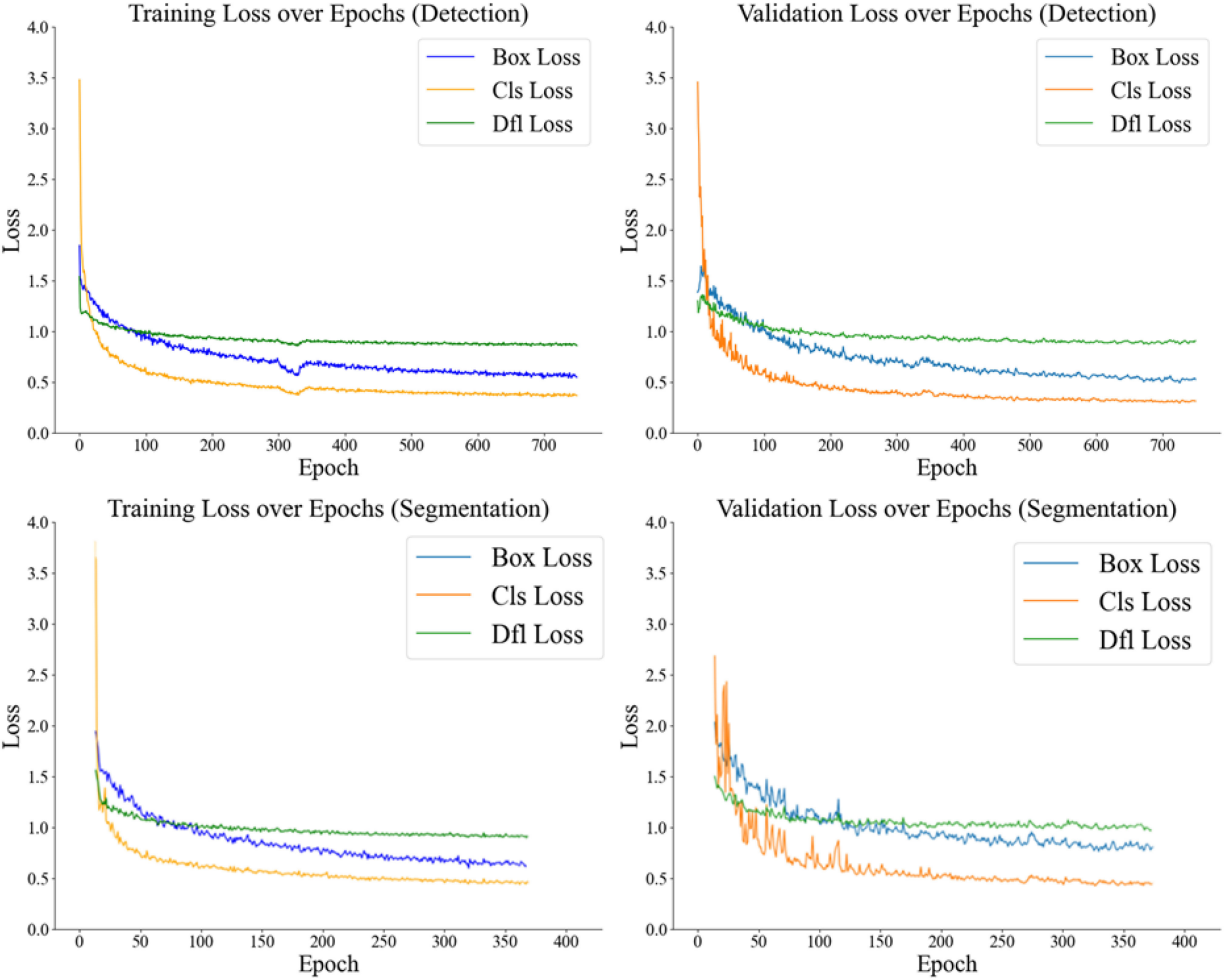
Changes in training and validation loss values over epochs for the YOLOv8 model trained for pest detection and segmentation.

In this study, significant improvements were observed during YOLOv8 model training for pest detection. For training metrics, the box_loss decreased from 1.84 to 0.54, cls_loss from 3.48 to 0.37, and dfl_loss from 1.54 to 0.86. Similarly, validation metrics showed a decline: val/box_loss reduced from 1.38 to 0.53, val/cls_loss from 3.45 to 0.31, and val/dfl_loss from 1.30 to 0.90. Training was halted at 749 epochs to prevent overfitting, demonstrating effective learning and performance. For segmentation, the train/box_loss decreased from 1.97 to 0.57, train/cls_loss from 4.04 to 0.37, and train/dfl_loss from 1.56 to 0.86, while validation metrics also improved, with val/box_loss reducing from 1.99 to 0.74, val/cls_loss from 2.65 to 0.37, and val/dfl_loss from 1.45 to 0.92. Training stopped at 372 epochs to avoid overfitting, indicating robust model performance (**Figure 3**).

### Performance evaluation metrics

**Figure 4:**
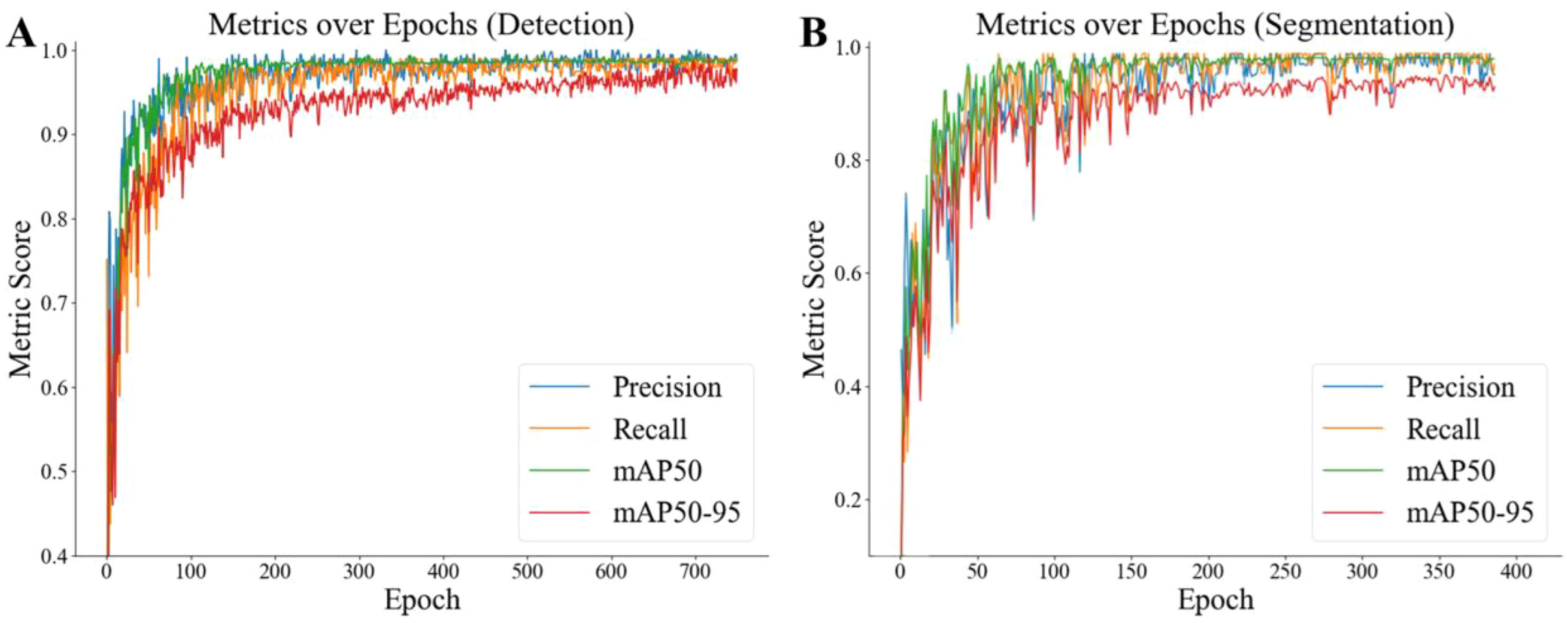
Improvements in performance metrics during YOLOv8 model training for object detection **(A)** and object segmentation **(B)**.

During YOLOv8 model training, significant improvements were noted across key metrics: precision increased from 0% to 98.91%, recall from 0% to 98.98%, mAP50 from 0% to 98.75%, and mAP50-95 from 0% to 97.72%. Training was halted at 749 epochs to prevent overfitting, demonstrating enhanced accuracy and reliability in object detection (**Figure 4A**). For segmentation, precision improved from 0% to 97.47%, recall from 0% to 98.81%, mAP50 from 0% to 99.38%, and mAP50-95 from 0% to 95.99%, with training stopping at 372 epochs to avoid overfitting (**Figure 4B**).

### Confusion matrix analysis

**Figure 5:**
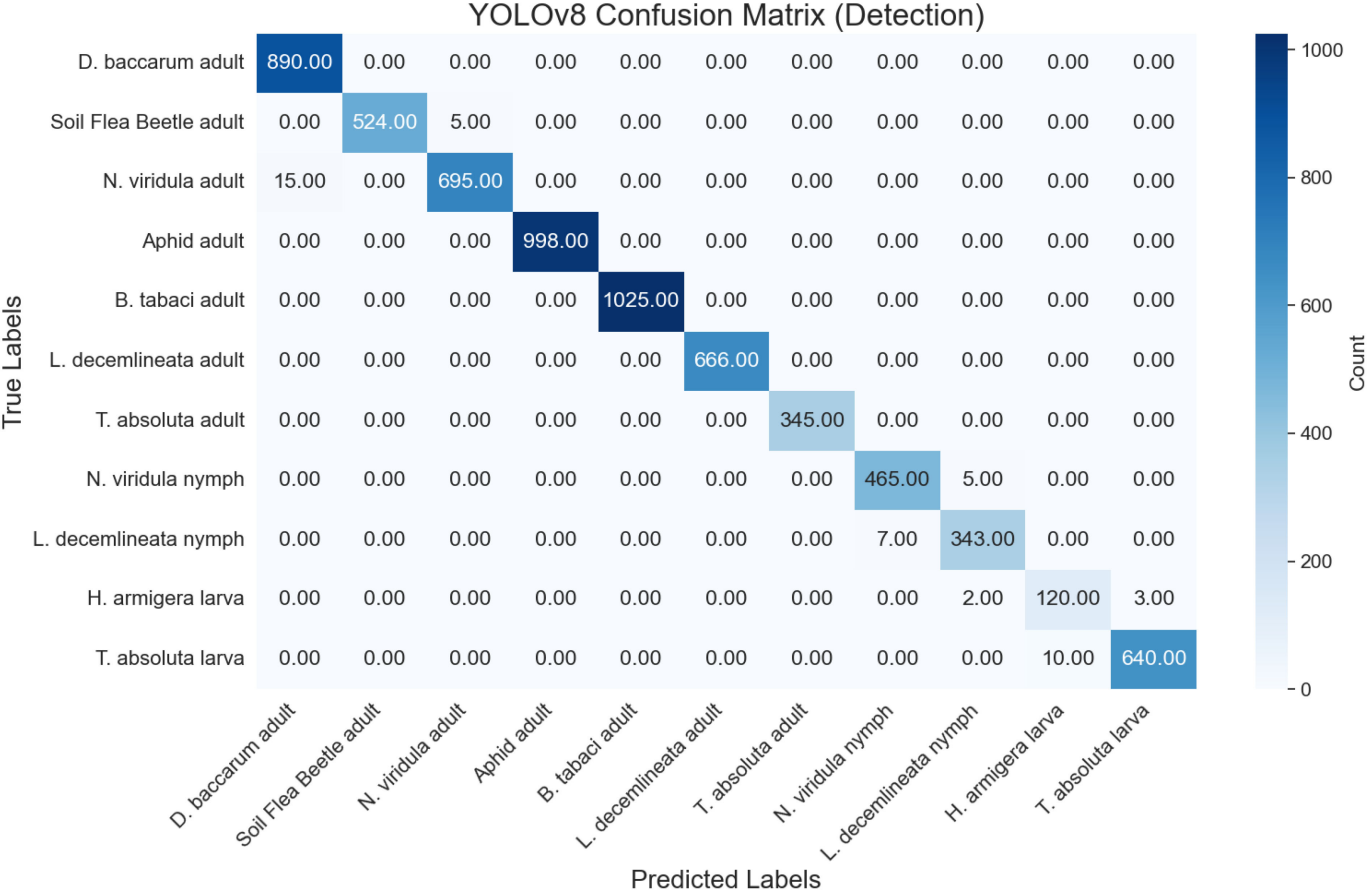
Confusion matrix illustrating YOLOv8 model’s performance in pest detection across 11 classes.

**Figure 6:**
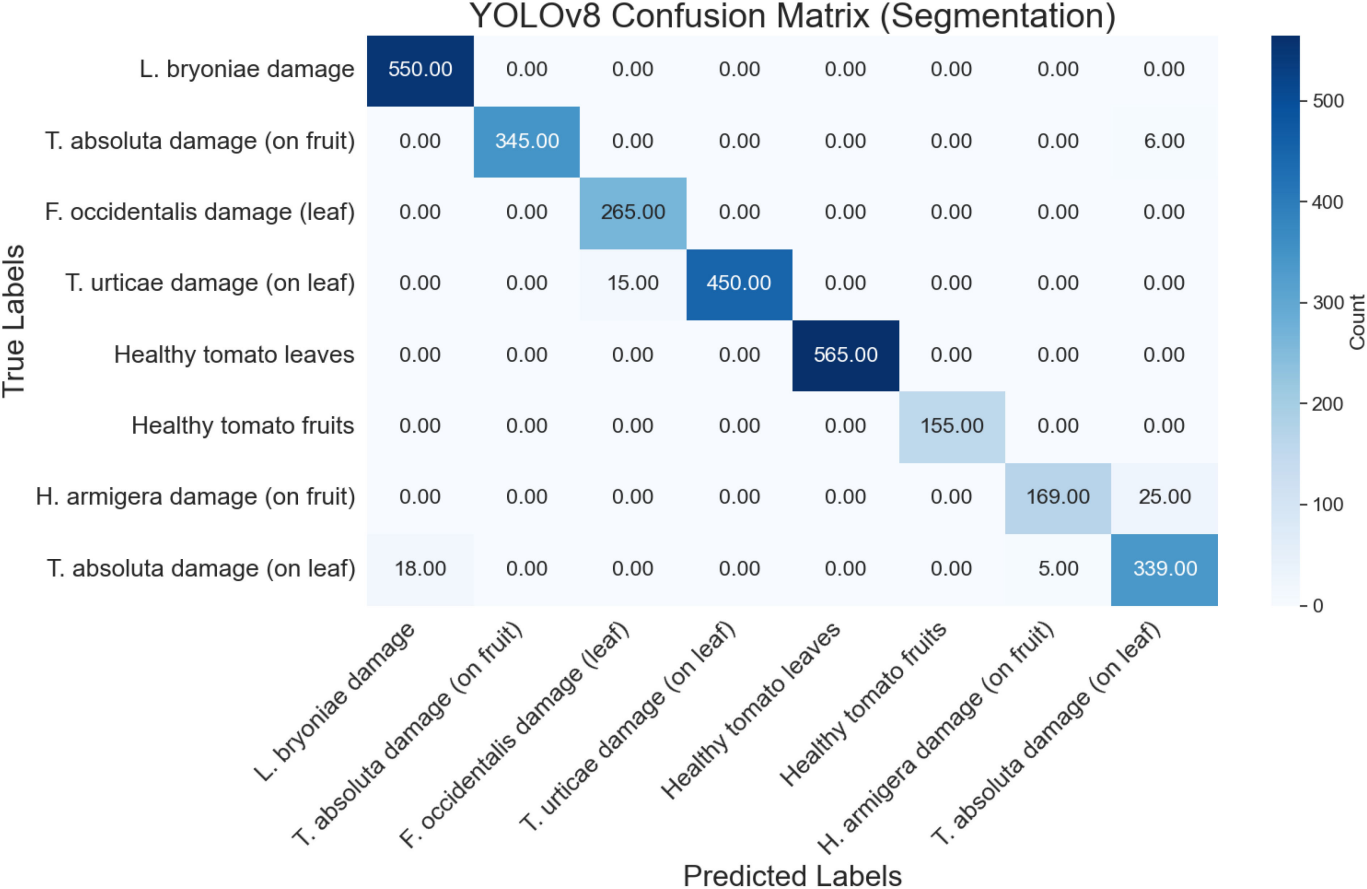
Confusion matrix illustrating YOLOv8 model’s performance in pest segmentation across eight classes.

A confusion matrix, essential for evaluating a model’s performance, pinpoints misclassifications and highlights areas for potential improvement. The test set, comprising images of adult insects, nymphs, and larvae across 11 classes, facilitated the computation of the confusion matrix. The YOLOv8 model exhibited high accuracy in detection tasks. Specifically, *D. baccarum* adults were correctly classified 890 times with 15 misclassifications, *N. viridula* adults were accurately identified 695 times with 15 errors, and *M. persicae* adults were correctly classified 998 times. Additionally, *B. tabaci* adults achieved 1025 correct identifications, and *L. decemlineata* adults were correctly identified 666 times with no errors (**Figure 5**). The model’s performance for segmentation on the test set revealed notable outcomes across 8 classes: 550 correct detections of *L. bryoniae* damage, 345 accurate detections of *T. absoluta* damage on fruit, 450 precise detections of *T. urticae* damage on leaves, and 565 correct identifications of healthy tomato leaves (**Figure 6**).

### Prompt creation and real-time textual response to visual data

**Figure 7:**
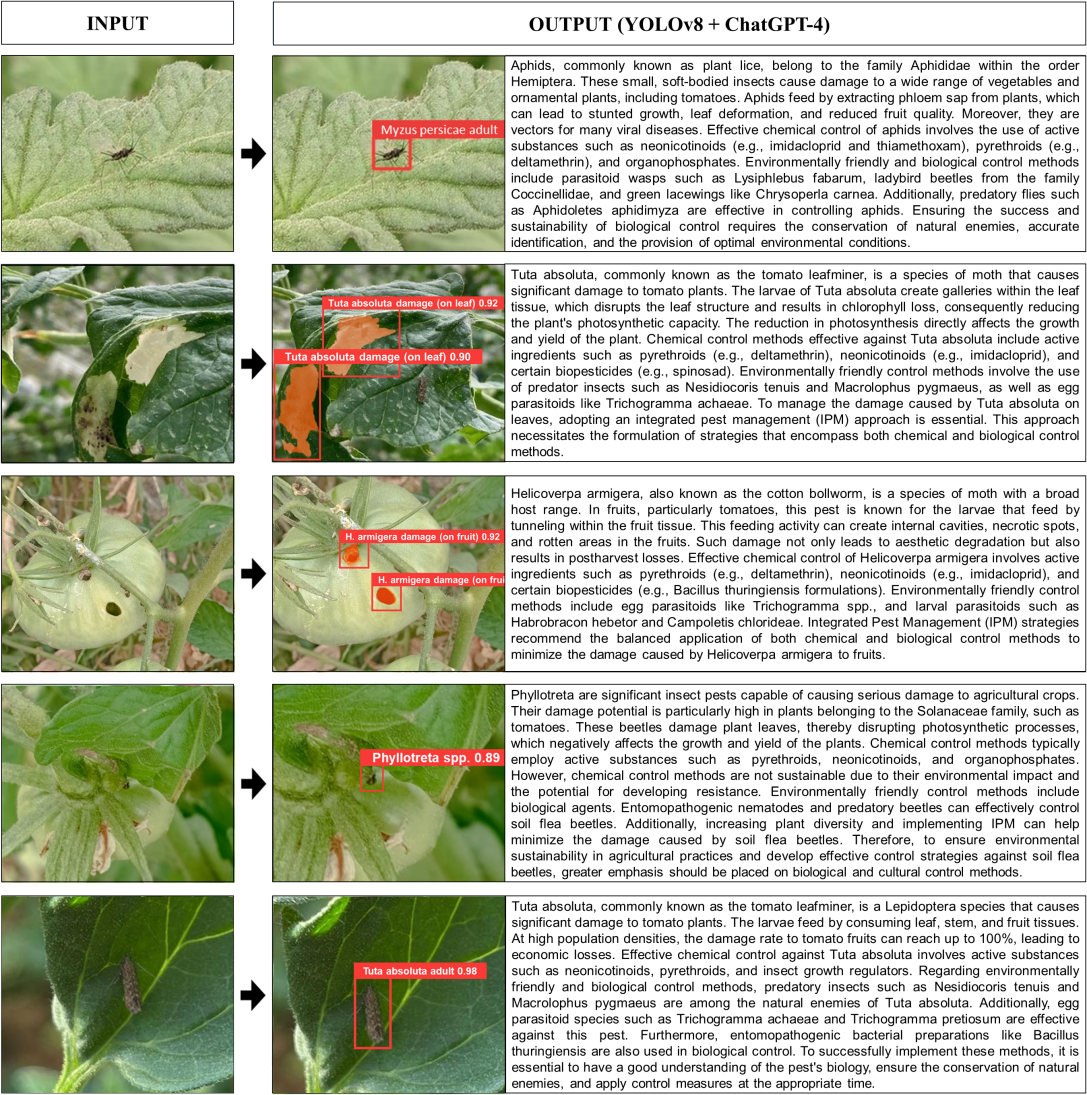
Feedback from ChatGPT-4 based on object labels detected by YOLOv8.

The integration of YOLOv8 and ChatGPT-4 showcases the powerful combination of computer vision and natural language processing, enabling expert feedback on visual data. This integration was tested on five pictures from the test set, which were not included during the training phase. The responses to the crafted prompt, [prompt_str = f”Could you provide a detailed explanation, in academic English, on the methods for controlling {det_labels_str} or {seg_labels_str} and the potential damage they can inflict on related plants, including preventative measures and integrated pest management strategies?”] are presented in **Figure 7**. The trained detection and segmentation models processed the test images in approximately 0.10 seconds, while the integration with ChatGPT-4 provided textual responses within 3.5 seconds via the API. Despite being limited to 250-400 tokens, the ChatGPT-4 responses, while not always fully comprehensive, demonstrated the potential to offer key information.

## Discussion

In the literature, numerous detection models such as Mask R-CNN, SSD, Detectron, and MobileNet are capable of identifying objects in photographs using image processing techniques (He et al., 2017; Liu et al., 2016; Girshick et al., 2014; Howard, 2017). However, among these models, YOLOv8 is preferred in this study due to its superior performance in agricultural pest detection (Redmon et al., 2016; Bochkovskiy et al., 2020). These superior results can be attributed to several factors, including the large and diverse dataset used for training, YOLOv8’s advanced architecture which allows for real-time processing with high accuracy, and the application of optimized hyperparameters specific to agricultural pest detection. For the detection task of the YOLOv8, precision increased to 98.91%, recall to 98.98%, mAP50 to 98.75%, and mAP50-95 to 97.72%. For segmentation tasks, precision increased to 97.47%, recall to 98.81%, mAP50 to 99.38%, and mAP50-95 to 95.99%. These results are consistent with other studies, such as the Pest-YOLO model achieving 69.59% mAP and 77.71% recall, and another study using YOLOv8 for small pest detection in field crops reporting an mAP of 84.7% (Khalid et al., 2023). Additionally, a study on pest detection in strawberries using segmented image datasets achieved a pest detection rate of 91.93% and detection reliability of 83.41% (Choi et al., 2022).

The integration of AI-based detection models with language models like ChatGPT offers significant benefits in pest detection and environmentally friendly pest control (Gu et al., 2021). Traditional methods, such as literature reviews, are resource-intensive, whereas language models provide rapid interpretations within 3.5 seconds, as demonstrated in this research. Researchers emphasize ChatGPT’s potential to train producers and improve information access (Ray, 2023; Siche and Siche, 2023). However, challenges exist regarding output accuracy, which depends on the training data (Gaddikeri et al., 2023). Inaccurate training data can compromise response precision, highlighting the need for training with credible sources.

Open-source language models like LLAMA (Meta) (Touvron et al., 2023), GPT-Neo and GPT-J (EleutherAI) (Black et al., 2022), BERT (Hugging Face) (Devlin et al., 2019), and GPT-2 (OpenAI) (Radford et al., 2019) allow for training on local computers with specific, reliable, and targeted datasets. Nevertheless, even with these advanced models, their effectiveness is contingent upon the quality of the input data and their ability to generalize across diverse agricultural environments. Furthermore, the computational power of personal computers may be insufficient for effectively using these models (Brown et al., 2020). While this study employed the YOLOv8 model integrated with the broadly-informed GPT-4 via an API, utilizing models trained with domain-specific, reliable data could enhance the accuracy and reliability of outputs. Future work should focus on training with domain-specific, trustworthy sources to improve accuracy and applicability across various sectors.

## Conclusion

The integration of AI-based detection and language models in this study demonstrates a significant advancement in agricultural practices. By embedding these models into common devices like smartphones, even untrained producers can access real-time expert consultation, enabling immediate pest detection and sustainable pest control. This technology holds the potential to revolutionize agriculture, particularly in remote areas, by reducing costs and facilitating integration with unmanned vehicles for continuous monitoring.

The study’s results, showing substantial improvements in detection and segmentation precision, recall, and mAP metrics, underscore the efficacy of YOLOv8 in agricultural applications. Additionally, integrating language models like ChatGPT enhances the system’s capability by providing detailed explanations and recommendations based on detected pests. This combination allows for rapid, informed decision-making, improving pest management strategies.

Future work should focus on training these models with domain-specific, reliable data to further enhance their accuracy and applicability. Moreover, addressing the computational limitations of personal devices for running advanced models will be crucial for broader adoption. To fully realize the potential of this technology in low-income and remote agricultural settings, future work should focus on the development of energy-efficient models that can run on low-power devices and operate under limited connectivity conditions. Additionally, partnerships with local agricultural cooperatives could facilitate the dissemination and training required for widespread adoption. Ultimately, this integration promises to democratize information access, promoting a more resilient, informed, and environmentally conscious approach to farming.

## Data Availability

The datasets presented in this study can be found in online repositories. The names of the repository/repositories and accession number(s) can be found in the article/supplementary material.
